# Recent advances in the detection of repeat expansions with short-read next-generation sequencing

**DOI:** 10.12688/f1000research.13980.1

**Published:** 2018-06-13

**Authors:** Melanie Bahlo, Mark F Bennett, Peter Degorski, Rick M Tankard, Martin B Delatycki, Paul J Lockhart

**Affiliations:** 1 Population Health and Immunity Division, The Walter and Eliza Hall Institute of Medical Research, Parkville, Victoria, Australia; 2 Department of Medical Biology, The University of Melbourne, Parkville, Victoria, Australia; 3 Epilepsy Research Centre, Department of Medicine, The University of Melbourne, Heidelberg, Victoria, Australia; 4 Mathematics and Statistics, Murdoch University, Murdoch, Australia; 5 Bruce Lefroy Centre for Genetic Health Research, Murdoch Children’s Research Institute, Royal Children’s Hospital, Parkville, Victoria, Australia; 6 Victorian Clinical Genetics Services, Parkville, Victoria, Australia; 7 Department of Paediatrics, The University of Melbourne, Parkville, Victoria, Australia

**Keywords:** short-read sequencing, short tandem repeats, repeat expansion disorders, bioinformatics

## Abstract

Short tandem repeats (STRs), also known as microsatellites, are commonly defined as consisting of tandemly repeated nucleotide motifs of 2–6 base pairs in length. STRs appear throughout the human genome, and about 239,000 are documented in the Simple Repeats Track available from the UCSC (University of California, Santa Cruz) genome browser. STRs vary in size, producing highly polymorphic markers commonly used as genetic markers. A small fraction of STRs (about 30 loci) have been associated with human disease whereby one or both alleles exceed an STR-specific threshold in size, leading to disease. Detection of repeat expansions is currently performed with polymerase chain reaction–based assays or with Southern blots for large expansions. The tests are expensive and time-consuming and are not always conclusive, leading to lengthy diagnostic journeys for patients, potentially including missed diagnoses. The advent of whole exome and whole genome sequencing has identified the genetic cause of many genetic disorders; however, analysis pipelines are focused primarily on the detection of short nucleotide variations and short insertions and deletions (indels). Until recently, repeat expansions, with the exception of the smallest expansion (SCA6), were not detectable in next-generation short-read sequencing datasets and would have been ignored in most analyses. In the last two years, four analysis methods with accompanying software (ExpansionHunter, exSTRa, STRetch, and TREDPARSE) have been released. Although a comprehensive comparative analysis of the performance of these methods across all known repeat expansions is still lacking, it is clear that these methods are a valuable addition to any existing analysis pipeline. Here, we detail how to assess short-read data for evidence of expansions, reviewing all four methods and outlining their strengths and weaknesses. Implementation of these methods should lead to increased diagnostic yield of repeat expansion disorders for known STR loci and has the potential to detect novel repeat expansions.

## Introduction

Expansions of known short tandem repeats (STRs) have been identified as the sole cause of disease for several orphan diseases but also can contribute substantially to the pathogenic variant burden in polygenic disease. Fragile X syndrome (OMIM #300624), the most common inherited cause of intellectual disability and autism, is caused by expansions of a CGG repeat in the 5′ untranslated region of the gene encoding fragile X mental retardation 1 ( *FMR1*) on the X chromosome. Unaffected individuals usually have STR alleles with a repeat motif number between 6 and 54. Affected male individuals have more than 200 copies of the motif. Huntington’s disease (OMIM #143100), one of the most common dominant disorders in Caucasians (the prevalence is 5 out of 100,000) ^1^, is caused by an expansion of a CAG repeat in the coding sequence of the huntingtin gene ( *HTT*). Unaffected individuals have between 6 and 35 motif copy numbers in their genomic sequence, and affected individuals have more than 35 motifs. Further examples include an expansion of the hexamer GGGGCC in the intron of *C9orf72*, which can cause both amyotrophic lateral sclerosis and fronto-temporal dementia (FTDALS1, OMIM #105550) and contributes the highest genetic risk burden of any single locus to both of these disorders. Currently, there are about 30 known repeat expansions that cause human diseases and that vary in terms of supporting literature. Twenty-one of these, which cause neurological disorders, have well-documented normal and pathogenic allele size ranges and are summarized in Table 1. The table includes several important non-neurological repeat expansion disorders, including the CTG expansion in TCF4, which causes the complex eye disorder Fuchs’ endothelial corneal dystrophy ^2^. In a recent discovery, the cause of FAME1 was found to be a complex pentamer repeat, situated in the gene *SAMD12* ^3^. This repeat is not present in normal individuals ( Table 1).

**Table 1.** Detailed short tandem repeat loci information for neurological disorders.

Disease	Symbol	OMIM	Inheritance	Gene	Cytogenetic location	Type	Repeat motif	Normal range	Expansion range	Strand	Start hg19	Reference repeat number	TRF match, %	TRF indel, %	Reference STR size, base pairs
Huntington disease	HD	143100	AD	*HTT*	4p16.3	Coding	CAG	6–34	36–100+	+	3,076,604	21.3	96	0	64
Kennedy disease	SBMA	313200	X	*AR*	Xq12	Coding	CAG	9–35	38–62	+	66,765,159	33.3	86	9	103
Spinocerebellar ataxia 1	SCA1	164400	AD	*ATXN1*	6p23	Coding	CAG	6–38	39–82	−	16,327,865	30.3	95	0	91
Spinocerebellar ataxia 2	SCA2	183090	AD	*ATXN2*	12q24	Coding	CAG	15–24	32–200	−	112,036,754	23.3	97	0	70
Machado-Joseph disease	SCA3	109150	AD	*ATXN3*	14q32.1	Coding	CAG	13–36	61–84	−	92,537,355	14	84	0	42
Spinocerebellar ataxia 6	SCA6	183086	AD	*CACNA1A*	19p13	Coding	CAG	4–7	21–33	−	13,318,673	13.3	100	0	40
Spinocerebellar ataxia 7	SCA7	164500	AD	*ATXN7*	3p14.1	Coding	CAG	4–35	37–306	+	63,898,361	10.7	100	0	32
Spinocerebellar ataxia 17	SCA17	607136	AD	*TBP*	6q27	coding	CAG	25–42	47–63	+	170,870,995	37	94	0	111
Dentatorubral- pallidoluysian atrophy	DRPLA	125370	AD	*DRPLA/* *ATN1*	12p13.31	Coding	CAG	7–34	49–88	+	7,045,880	19.7	92	0	59
Huntington disease-like 2	HDL2	606438	AD	*JPH3*	16q24.3	Exon	CTG	7–28	66–78	+	87,637,889	15.3	95	4	47
Fragile-X site A	FRAXA	300624	X	*FMR1*	Xq27.3	5′ UTR	CGG	6–54	200–1,000+	+	146,993,555	25	90	5	75
Fragile-X site E	FRAXE	309548	X	*FMR2*	Xq28	5′ UTR	CCG	4–39	200–900	+	147,582,159	15.3	100	0	46
Myotonic dystrophy 1	DM1	160900	AD	*DMPK*	19q13	3′ UTR	CTG	5–37	50–10,000	−	46,273,463	20.7	100	0	62
Friedreich ataxia	FRDA	229300	AR	*FXN*	9q13	Intron	GAA	6–32	200–1,700	+	71,652,201	6.7	100	0	20
Myotonic dystrophy 2	DM2	602668	AD	*ZNF9/CNBP*	3q21.3	Intron	CCTG	10–26	75–11,000	−	128,891,420	20.8	92	0	83
Frontotemporal dementia and/or amyotrophic lateral sclerosis 1	FTDALS1	105550	AD	*C9orf72*	9p21	Intron	GGGGCC	2–19	250–1,600	−	27,573,483	10.8	74	8	62
Spinocerebellar ataxia 36	SCA36	614153	AD	*NOP56*	20p13	Intron	GGCCTG	3–8	1500–2,500	+	2,633,379	7.2	97	0	43
Spinocerebellar ataxia 10	SCA10	603516	AD	*ATXN10*	22q13.31	Intron	ATTCT	10–20	500–4,500	+	46,191,235	14	100	0	70
Myoclonic epilepsy of Unverricht and Lundborg	EPM1	254800	AR	*CSTB*	21q22.3	Promoter	CCCCGCCCCGCG	2–3	40–80	−	45,196,324	3.1	100	0	37
Spinocerebellar ataxia 12	SCA12	604326	AD	*PPP2R2B*	5q32	Promoter	CAG	7–45	55–78	−	146,258,291	10.7	100	0	32
Spinocerebellar ataxia 8	SCA8	608768	AD	*ATXN8OS/* *ATXN8*	13q21	utRNA	CTG	16–34	74+	+	70,713,516	15.3	100	0	46
Spinocerebellar ataxia 31	SCA31	117210	AD	*BEAN1/TK2*	16q21	Intron	TGGAA ^a^	N/A	2.5–3.8 kb ^b^	+	66,524,302	0	N/A	N/A	N/A
Spinocerebellar ataxia 37	SCA37	615945	AD	*DAB1*	1p32.2	Intron	ATTTC ^a^	0	31–75	−	57,832,716 ^c^	0	N/A	N/A	N/A
Familial adult myoclonic epilepsy 1 ^d^	FAME1	601068	AD	*SAMD12*	8q24	Intron	TTTCA ^a^	0	440–3,680 ^e^	−	119,379,055 ^c^	0	N/A	N/A	N/A
Fuchs endothelial corneal dystrophy 3	FECD3	613267	AD	*TCF4*	18q21.2	Intron	CTG	10–40	50–150+	−	53,253,385	25.3	100	0	76
Oculopharyngeal muscular dystrophy	OPMD	164300	AD	*PABPN1*	14q11.2	Coding	GCG	6–7	8–13	+	23,790,682	6.7	100	0	20
Early infantile epileptic encephalopathy 1 ^f^	EIEE1	308350	X	*ARX*	Xp21.3	Coding	GCG	7–12 ^f^	17–20 ^f^	−	25,031,771	14.7	90	0	44

Detailed short tandem repeat (STR) loci information for disorders associated with repeat expansions. Tandem Repeats Finder (TRF) (Benson ^16^ 1999) match and TRF indel describe the purity of the repeat. AD, autosomal dominant; AR, autosomal recessive; N/A, not applicable; UTR, untranslated region; X, X-linked. ^a^As these repeats are insertions, the motifs do not appear in the reference at the respective locus. ^b^SCA31 is caused by the insertion of a complex repeat containing (TGGAA) _n_; thus, the base-pair length of expanded repeats is given instead of repeat number. ^c^The SCA37 position is given at the reference (ATTTT) _n_ repeat, of which affected individuals have (ATTTC) _n_ inserted. The FAME1 position is given at the reference (TTTTA) _n_ repeat, of which affected individuals have (TTTCA) _n_ inserted. ^d^Ishiura *et al*. ^3^ identified similar expansions associated with FAME6 and FAME7 but only in single families. The same TTTCA repeat insertion was observed in the intronic region of *TNRC6A* and *RAPGEF2*, respectively. ^e^The size of the FAME1 repeat is the estimated combined size of the expanded (TTTCA) _n_ insertion and (TTTTA) _n_ reference repeat. ^f^Different polyalanine expansions in the gene can be expanded.

Repeat expansion tests are instigated by clinicians in response to a suspected clinical diagnosis. Detection of repeat expansions is performed by using methods such as polymerase chain reaction (PCR) for the shorter repeat expansions or Southern blot for longer repeats. Repeat expansion locus-specific PCR methods, such as repeat-primed PCR ^4^, have also been developed by individual laboratories and represent an active area of research in diagnostic methods ^5^. These methods are also able to accurately size repeat expansions.

Genetic laboratories conduct a large number of tests for repeat expansion disorders, but the detection rate is low. Turnaround times are of the order of weeks or months. No comprehensive panel or testing method exists that simultaneously tests for all known repeat expansions using the current gold-standard detection methods of PCR and Southern blot.

Next-generation sequencing (NGS), with either whole exome (WES) or whole genome (WGS) sequencing, is now a standard test for many individuals with a suspected genetic disorder. DNA sequencing analysis is a highly streamlined process that can be outsourced to one of the many clinically accredited sequencing laboratories worldwide. The analysis is performed by using sophisticated pipelines ^6^. Even with outsourced data, results are often delivered within a few weeks. If analysis is performed in-house, turnaround could be as fast as a week for WGS and a few days for WES, depending on the computer capacity available.

Analysis of WES and WGS data is very efficient in the identification of single-nucleotide variants and indels but also can examine structural variation, such as copy number variation. Standard variant pipelines report mismatches of up to 50 base pairs (bp) ^7^ and thus can identify only short STR alleles ^8^. Furthermore, these are often poorly described in the variant call format output files. Some improvements in the identification of STR variants came from larger indel detection methods such as DINDEL ^9^ and PINDEL ^10^. Since 2000, several methods have also sought to specifically identify the lengths of STR alleles from short-read NGS data. One of the most recent methods is HipSTR ^11^, which uses an Expectation Maximization (EM) algorithm to determine the set of STR alleles present at a locus. The EM algorithm is combined with a local realignment step, and was found to outperform existing methods. However, all of these methods are constrained to STR alleles with repeat lengths smaller than the read length employed in the sequencing. Standard WGS short-read sequencing for the highest throughput sequencing platform, the Illumina HiSeq X Ten, uses a paired-end protocol with reads of 150 bp in length. WES is now also performed by using paired-end sequencing with reads of 150 bp; however, some data sets—in particular, older data sets—have shorter read lengths. Hence, many of the repeat expansion alleles that cause disease remain undetectable by these standard pipeline variant-calling methods. The ability to detect known and possibly novel repeat expansions with short-read sequencing data would be a valuable addition to any clinical genomics or diagnostic sequencing pipeline.

Four new methods to detect repeat expansions have recently been described: ExpansionHunter ^12^, exSTRa ^13^, STRetch ^14^, and TREDPARSE ^15^. All four have demonstrated the ability to detect repeat expansions where the expanded allele size is greater than the length of standard short-read sequencing reads and even the read pair fragment length. In this review, we briefly outline the principles behind these methods, comparing their approaches. By introducing these methods, we hope to encourage researchers and clinical genomics facilities to incorporate them into their pipelines, as we believe it will improve molecular genetic diagnosis with the greatest impact to be expected for neurological disorders. We also discuss applications of these approaches beyond clinical genomics and finish with some comments regarding the potential of the developing long-read sequencing technologies for the detection of expanded alleles.

## How to detect repeat expansions with short-read data

**Figure 1.**
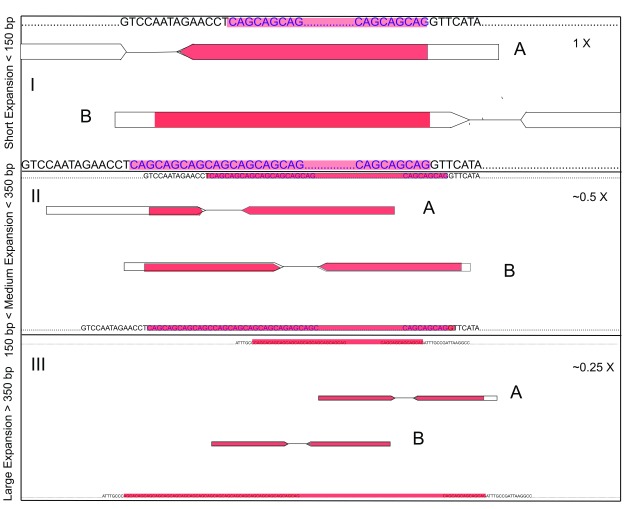
Detecting repeat expansions with short-read sequencing data. Depicted are three scenarios: ( **I**) a short repeat expansion where the repeat expansion is less than 150 base pairs (bp), or smaller than a read; ( **II**) a medium-size repeat expansion where the repeat expansion is between 150 and 350 bp; and ( **III**) a large repeat expansion, where the repeat expansion is greater than 350 bp. For each of the three panels, **I**– **III**, the top line of DNA sequence depicts the reference sequence, and the bottom line depicts the (not known) repeat expansion size sequence. Red segments in reads signify repeat sequence. Evidence from reads varies according to the repeat size. For all three scenarios, there is information in reads that map into the repeat (A) but for scenario **I** occasional reads span the expanded allele (B), giving information about the size of the expanded allele. In scenario **II**, some read fragments can span expanded allele and can also be used for inference. For large expansions, some read fragments stem entirely from the expanded alleles. These may not be unambiguously mapped and are exploited only by ExpansionHunter (large motifs only) and TREDPARSE (based on fragment size information).

The repeat expansion detection methods discussed here all require paired-end sequencing data. Standard paired-end sequencing provides a pair of reads that flank a DNA fragment of about 350 bp in length. Library preparations can vary this DNA fragment size, and larger fragments are known to be advantageous for applications such as genome assembly, which could also be potentially useful for expansion detection. The two reads that comprise a read pair are sequenced in opposite directions, toward each other. Between the read pairs, there is typically a short sequence of DNA (of about 50 bp in length) that is not sequenced. The key to repeat expansion detection is to assess reads that are found to lie partially, or entirely, in an STR for their repeat content. This can be done heuristically (Expansion, exSTRa, and STRetch) or can be integrated into a likelihood model (TREDPARSE). Expanded alleles at an STR will contribute reads with more repeat content and more reads in total than reads stemming from the normal, unexpanded, allele ( Figure 1).

Key factors that will influence the ability to detect repeat expansions are (i) the library preparation protocol, (ii) the read length and likely also the DNA fragment length, and (iii) the depth of sequencing employed for the sample. These factors influence the number of reads that cover each STR locus. Tankard *et al*. ^13^ compared several library preparation protocols over the 21 known neurological STR loci, showing locus-specific effects for these factors (Supplementary Figure 1). In general, PCR-free WGS library preparation protocols yield the best data to allow repeat expansion detection, but even WES data could be successfully interrogated for repeat expansions for most of the known repeat expansion loci captured during library preparation ^13^.

exSTRa and ExpansionHunter determine the repeat content of all reads mapped to a particular STR locus. This then forms the source data for their respective analyses. TREDPARSE includes the repeat content into its likelihood model and estimates the repeat motif number, similarly to HipSTR and lobSTR ^17^. For large expanded repeats, it is possible that entire DNA fragments lie within the STR. The paired-end reads that capture only repeat content either map to other regions in the genome where longer copies of this repeat are present in the genomic reference or remain unmapped for both reads of the read pair ( Figure 1). ExpansionHunter labels these reads as in-repeat reads, or IRRs, whereas exSTRa, STRetch, and TREDPARSE discard them from analysis. If the motif is long and sufficiently under-represented in the genome, as is the case with the hexamer GGGGCC *C9orf72* expansion ( Table 1), then there will be only a small number of alternate locations where reads containing the expanded allele could preferentially map to instead of the original locus. ExpansionHunter assesses the additional 29 sites with larger copy numbers of this hexamer repeat and incorporates this information into its likelihood to estimate the allele sizes of the individual.

STRetch employs a different approach, whereby a new reference genome is proposed with additional decoy chromosomes containing artificially long versions of all repeat motif combinations. The decoy chromosomes provide an alternative mapping location for the expanded reads. This method requires the initially computationally expensive step of realignment to a new reference genome but results in a very natural statistical testing framework where relative read alignment between a candidate STR and its decoy are compared in a likelihood ratio test. However, it requires that the decoy chromosomes encode repeat motif representations that ensure that the reads of the expanded STR allele preferentially map there. Short repeat expansions, such as SCA6, where expanded alleles have as few as 21 repeat motifs, may preferentially map as insertions at their original location rather than to the (longer) expansion in the decoy chromosome, remaining undetected. In contrast, older, within-read only detection methods, such as lobSTR, can detect such expansions.

ExpansionHunter and exSTRa do not require additional alignment steps. Instead, they interrogate existing alignments. STRetch requires re-alignment to the augmented reference genome, although alignment can potentially be performed in an *ad hoc* manner by taking reads that have failed to align with the standard genome reference and aligning these solely to the set of decoy chromosomes. The effects of this approach have not yet been evaluated. TREDPARSE also has a potentially time-consuming local realignment step similar to HipSTR ^11^, which has yet to be evaluated in a genome-wide analysis. We refer the reader to each of the four articles for depictions of the types of read evidence that are used in each of the algorithms.

The possibility of an expansion is assessed differently for each of the methods. exSTRa and STRetch rely on the availability of controls to allow them to determine whether an individual is an outlier with respect to their statistical measures. ExpansionHunter and TREDPARSE can be used on a single sample for known loci, making use of known thresholds and empirical distribution properties in the STR allele size to identify individuals with expansions. For novel repeat expansion loci, appropriate thresholds are not known and will require *post hoc* testing of the allele size distribution in a control cohort to assess likely outlier individuals. This latter test is not currently implemented in ExpansionHunter or TREDPARSE.

TREDPARSE makes use of a highly parameterized likelihood framework with a stuttering model and a local realignment step to infer allele sizes and determine the likelihood of pathogenicity. ExpansionHunter employs a much simpler likelihood model, which infers allele sizes and then uses allele thresholds to determine significance. STRetch applies a likelihood ratio test comparing the relative mapping of the reads for a known repeat to the expected genomic location or to its decoy chromosome containing that repeat. exSTRa uses a simple summary statistic combined with an outlier detection method and, like STRetch, uses a set of controls to apply permutation testing to assess the significance of the findings. Despite the variety of evaluation frameworks and statistical approaches, ExpansionHunter, exSTRa, and STRetch were able to detect almost all of the known repeat expansions that they were tested on whereas TREDPARSE was found to produce results that were validated with alternative methods such as long-read sequencing. We refer readers to the respective articles for details of the variety of performance evaluations that have been employed by these methods. We summarize the properties of the four algorithms in Table 2.

**Table 2.** Summary of computational methods, evaluation framework, and limitations for ExpansionHunter, exSTRa, STRetch, and TREDPARSE.

Software	Publication	Computational burden ^a^: known loci/ genome-wide	Statistical test	Reported WGS/WES analysis capability	Software ease of use	Ability to search genome- wide	Graphical output	Length of STR expansion detection bias
ExpansionHunter	Dolzhenko *et al*. ^12^, *Genome* *Research* 2017	Low/Low	None – estimates allele sizes. Significance determined on the basis of thresholds ^b^.	WGS	High	Possible	No	Repeats with long motifs (e.g., c9orf72 ^c^) gain extra evidence for expansion with usage of in-repeat reads (IRRs)
exSTRa	Tankard *et al*. ^13^, bioRxiv, 2017	Low/Medium	Permutation based outlier detection test	WGS and WES	Medium	Possible	Yes	No known bias
STRetch	Dashnow *et al*. ^14^, bioRxiv, 2017	High/Medium	Likelihood ratio test with reads mapping to decoy. Estimates allele sizes.	WGS	Low	Easy	No	Short expansions may not map to the decoy chromosomes and remain undetected (e.g., SCA6 ^d^)
TREDPARSE	Tang *et al*. ^15^, AJHG, 2017	Low/Unknown	Likelihood of pathogenicity, genetic model, estimates allele sizes ^b^	WGS	High	Possible	Yes	Does not detect expansions that exceed its detection threshold (300 repeats)

^a^Computational burden has been split into two components: known loci—a small subset of all short tandem repeat (STR) loci—and genome-wide, representing thousands of STR loci. ^b^Requires prior information for STR in terms of allele size to aid statistical test. ^c^The C9orf72 repeat expansion is a hexamer repeat. ^d^SCA6 is the smallest repeat expansion currently known. WES, whole exome sequencing; WGS, whole genome sequencing.

## The role of known repeat expansions in related disorders such as epilepsy

Several genes that contain disease-causing repeat expansions that cause ataxias have been implicated in other disorders such as epilepsy and migraine. For example, the clinical spectrum of *C9orf72* has broadened to encompass Huntington’s disease-like disorder ^18^. The new repeat expansion detection methods described here permit an investigation of the role of all known repeat expansions in cohorts of individuals with related phenotypes, such as epilepsy and migraine. These can be examined using existing short-read data with the new repeat expansion detection methods, potentially providing new insights.

## Evaluating the variation of repeat expansion short tandem repeats in cohorts

STRs vary in frequency and length distributions between different ethnic groups because of founder effects. Willems *et al*. ^11^ used lobSTR to generate an online STR catalogue ( http://strcat.teamerlich.org) of about 700,000 STRs, which displays STR repeat number distributions and, where possible, stratification by the 14 ethnicities represented in the 1000 Genomes project ^19^. Many STR loci were found to display ethnicity-specific distributions. Several repeat expansion STRs also show ethnicity effects. For example, the CAG repeat in the ataxin 7 gene ( *ATXN7*) displays multiple founder events in Scandinavia, Mexico, and South Africa/Zimbabwe ^20^, and multiple founder events have also been documented for Huntington’s disease ^1^.

The remarkable reduction in the price of short-read sequencing has led to the sequencing of greater numbers of individuals and new study cohorts. By making use of the methods reviewed here, analyses of the genetic composition of pathogenic STR loci in hitherto unexamined cohorts will be possible. An understanding of the natural variation of both normal and repeat expansion alleles in different populations will also be helpful to refine the statistical tests of the methods, including providing more accurate information for the determination of significance thresholds and prior information required for testing.

Prospective and retrospective analysis of sequencing data sets with the repeat expansion detection methods described here should provide clinically actionable outcomes. Also exciting are the research opportunities in our understanding of STRs. For example, spinocerebellar ataxia-8 (SCA8, OMIM #608768) is one of several poorly understood disorders caused by a repeat expansion ^21^. The repeat is bidirectionally transcribed ^22^. Additionally, its clinical implications are still uncertain, and the understanding of its clinical spectrum and penetrance is incomplete. Using large population-based and disease-ascertained cohorts containing thousands of individuals, we will be able to gather hundreds of detected repeat expansions for these repeats, allowing a more precise determination of penetrance and potential co-morbidities. To determine proof of principle, Tang *et al*. ^15^ profiled 12,632 individuals, identifying 132 individuals with larger-than-normal-range STR alleles at 15 different known repeat expansion STR loci.

## Detecting novel repeat expansions

It is likely that novel repeat expansion loci are awaiting discovery. In OMIM, there are several reported SCA loci, such as SCA32 (OMIM %613909, 7q32-q33), that as yet have no determined genetic cause. Families with linkage to SCA25 ^23, 24^ (OMIM %608703, 2p21-p13) furthermore report the phenomenon of anticipation. Anticipation is a hallmark of repeat expansions since these can become more unstable (and usually larger) with subsequent meioses, after the initial expansion step, thus leading to earlier ages of onset or more severe symptoms (or both) in affected individuals from more recent generations in the pedigree.

ExpansionHunter, TREDPARSE, exSTRa, and STRetch are all able to detect novel repeat expansions but require that the putative expansion STRs be explicitly specified. Hence, all methods rely on *a priori* knowledge of STR loci to be examined. STR sets of interest can be assembled by using annotation of STRs from Tandem Repeats Finder results ^16^ and appropriate search parameters. Relevant parameters such as motif length, existing repeats, and purity of the repeat will determine the number of STRs detected in the reference genome being examined. The expansion detection performance of methods will be influenced by the genomic composition of the STRs, with complex STRs, with features such as impure repeats or multiple repeat motifs comprising a single STR likely to be more difficult to detect.

## Implementation limitations

Although all four of the methods discussed (TREDPARSE, exSTRa, ExpansionHunter, and STRetch) will benefit from further development, we advocate the immediate implementation of these methods to any existing analysis pipelines for WES, WGS, or even suitable gene panels. The initial benefit will be through the examination of retrospective and prospectively sequenced individuals for all known repeat expansion loci to prevent a missed diagnosis due to a known expansion ^18, 25^. These missed molecular diagnoses are an important contributor to increasing diagnostic yield in clinical genomic sequencing ^26^. Individuals detected to have a repeat expansion with one, or more, of ExpansionHunter, TREDPARSE, exSTRa, or STRetch should undergo the gold-standard assays at a certified laboratory, when possible, or at a research laboratory specializing in the repeat expansion detection of that STR locus.

Although all four publications describe the application of the methods to a variety of known repeat expansions, none of them encompasses a complete list of known loci. Indeed, at the moment, there are several repeat expansion loci that have never been tested with any of the four computational approaches. These include EPM1 ( *CSTB*, OMIM #254800), HDL2 ( *JPH3*, OMIM #606438), SCA10 ( *ATXN10*, OMIM #603516), and SCA12 ( *PPP2R2B*, OMIM #604326). It is likely that the algorithms will be able to efficiently interrogate most or all of these loci, similar to the majority of other repeat expansion loci that have been tested. All four of these loci achieve good coverage with PCR-free WGS protocols ^13^.

Some STR loci such as FRAXA ( *FMR1*, OMIM #300624) are highly adversely affected by PCR amplification bias introduced during library preparation and could be assessed only with a PCR-free library preparation protocol ^13^. FRAXE ( *FMR2*, OMIM #309548) remains refractory to capture with short-read sequencing, regardless of the protocol used, and is not currently assessable with any of the repeat expansion detection methods.

## The continuing role of gold-standard repeat detection methods such as Southern blots and repeat-primed polymerase chain reaction

Repeat expansion detection methods such as Southern blots, PCRs, and repeat primed PCR will not be supplanted soon, even with these developments in the detection of repeat expansions with NGS. First, the latter should be seen as a screening method, requiring validation with the gold-standard methods. Second, the NGS-based methods cannot, as yet, accurately and reliably size repeat expansions. Furthermore, it is unlikely that the short-read methods will be able to do so, even in the future, since they rely on imperfect relationships between read numbers and repeat allele length, which is more difficult for larger repeats.

Comprehensive prospective studies will also be needed to compare the cost and efficacy of the NGS-based methods for screening for repeat expansions before NGS-based screening approaches are adopted.

Long repeat expansion alleles (>500 bp), such as those found in DM1 ( *DMPK*, OMIM #160900), FRAXA, FRAXE, and SCA10 patients, are difficult to detect with standard diagnostic tests. The use of NGS-based detection of repeat expansions could improve overall diagnostic yield for these ultra-long expansions since these methods have been demonstrated to perform well for loci such as DM1 and FRDA ( *FXN*, OMIM #229300). NGS-based repeat expansion detection may also be more accessible for some patients than current gold-standard methods because NGS is a commonly used, robust platform, which has seen a continuing drop in costs and even wider availability.

## The impact of long-read sequencing

Although advances in repeat expansion detection with short-read sequencing are exciting, the next wave of discovery, owing to the increased quality and rapidly decreasing costs of long-read sequencing, is already upon us. Long-read sequencing technologies such as PacBio and Nanopore sequencing are rapidly gaining popularity and attracting significant bioinformatics interest to improve analysis pipelines. The reported read lengths are in the tens of thousands of base pairs rather than the hundreds. As such, these long-read sequencing platforms will sequence through STR loci for both normal and expanded alleles. This will be particularly useful for complex expanded alleles, where the repeat may be interrupted multiple times. Neither NGS-based methods nor current diagnostics methods do well in these cases. Sequencing error rates are currently still much higher for long-read sequencing than for short-read sequencing and will require further work to be able to reliably determine repeat lengths ^27^. Nanopore sequencing has the additional advantage of having no GC coverage bias because there is no DNA polymerization step ^28^. GC bias in repeats or flanking regions (or both) can lead to a bias in allele amplification with bias observed both for and against the expanded allele ^13^.

Further novel repeat expansions are doubtlessly awaiting discovery. Their discovery will be aided by novel analytical approaches such as those reviewed here. They will likely require support from other sequencing methods, including long-read sequencing and RNA-seq ^26^. The biological mechanisms underpinning these diseases are a separately fascinating and rapidly broadening field of research. Additionally, new technologies are leading to renewed hope for potential treatments. A recent publication described the elimination of the toxic effect of the CTG expansion in DM1 (OMIM #160900) with RNA targeting Cas9 excision ^29^. This is an exciting time for research in repeat expansion disorders.
